# Molecular identification of *Proteus mirabilis*, *Vibrio species* leading to CRISPR-Cas9 modification of *tcpA* and *UreC* genes causing cholera and UTI

**DOI:** 10.1038/s41598-024-59340-9

**Published:** 2024-04-12

**Authors:** Muhammad Naveed, Fatima Tahir, Tariq Aziz, Muhammad Waseem, Syeda Izma Makhdoom, Nouman Ali, Metab Alharbi, Thamer H. Albekairi, Abdullah F. Alasmari

**Affiliations:** 1 Department of Biotechnology, Faculty of Science and Technology, University of Central Punjab, Lahore, 54590 Pakistan; 2 Laboratory of Animal Health, Food Hygiene and Quality, Department of Agriculture, University of Ioannina, 47100 Arta, Greece; 3 Department of Pharmacology and Toxicology, College of Pharmacy, King Saud University, P.O. Box 2455, 11451 Riyadh, Saudi Arabia

**Keywords:** Heavy metals, Cadmium resistance bacteria, Bioaccumulation, 16S rRNA amplification, CRISPR-Cas9, gRNA, UTI, Cholera, Biochemistry, Chemical biology, Computational biology and bioinformatics, Drug discovery, Immunology, Microbiology, Molecular biology

## Abstract

Heavy metal accumulation increases rapidly in the environment due to anthropogenic activities and industrialization. The leather and surgical industry produces many contaminants containing heavy metals. Cadmium, a prominent contaminant, is linked to severe health risks, notably kidney and liver damage, especially among individuals exposed to contaminated wastewater. This study aims to leverage the natural cadmium resistance mechanisms in bacteria for bioaccumulation purposes. The industrial wastewater samples, characterized by an alarming cadmium concentration of 29.6 ppm, 52 ppm, and 76.4 ppm—far exceeding the recommended limit of 0.003 ppm—were subjected to screening for cadmium-resistant bacteria using cadmium-supplemented media with CdCl_2_. 16S rRNA characterization identified *Vibrio cholerae* and *Proteus mirabilis* as cadmium-resistant bacteria in the collected samples. Subsequently, the cadmium resistance-associated cadA gene was successfully amplified in Vibrio species and Proteus mirabilis, revealing a product size of 623 bp. Further analysis of the identified bacteria included the examination of virulent genes, specifically the tcpA gene (472 bp) associated with cholera and the UreC gene (317 bp) linked to urinary tract infections. To enhance the bioaccumulation of cadmium, the study proposes the potential suppression of virulent gene expression through in-silico gene-editing tools such as CRISPR-Cas9. A total of 27 gRNAs were generated for UreC, with five selected for expression. Similarly, 42 gRNA sequences were generated for tcpA, with eight chosen for expression analysis. The selected gRNAs were integrated into the lentiCRISPR v2 expression vector. This strategic approach aims to facilitate precise gene editing of disease-causing genes (tcpA and UreC) within the bacterial genome. In conclusion, this study underscores the potential utility of Vibrio species and Proteus mirabilis as effective candidates for the removal of cadmium from industrial wastewater, offering insights for future environmental remediation strategies.

## Introduction

In the last century, accelerated urbanization driven by industrial growth has led to the release of diverse pollutants into the environment, posing a significant threat to ecosystems and human health^1^. Heavy metals, resulting from anthropogenic activities like metal mining, industrial production, and agricultural practices, constitute a prominent category of pollutants^2^. Noteworthy heavy metals, such as arsenic (As), chromium (Cr), cadmium (Cd), zinc (Zn), mercury (Hg), lead (Pb), thallium (Tl), and nickel (Ni), exhibit severe health impacts in elemental and combined forms^3,4^. Accumulation of these metals in vital organs, particularly the liver and gills of marine animals, highlights the pervasive nature of contamination in aquatic ecosystems. Prolonged exposure to heavy metals poses increased risks of renal and hepatic damage to industrial workers and residents near contaminated sites^5^. Cadmium, specifically, has garnered attention for its adverse health effects, emphasizing the need for targeted research and remediation strategies to address its contamination in water sources and agricultural products^6^. Understanding the impact of cadmium is crucial for developing effective measures to safeguard human health and the environment.

The safe cadmium limit for wastewater and soil is 0.003 ppm recommended by WHO. The exceeded limits can cause respiratory problems, lung and renal damage, prostate cancer, and skeletal and cardiovascular problems in children^7^. The study was conducted in Nairobi, Kenya, to assess the levels of heavy metals in wastewater and soil. The cadmium concentration was 0.000087 ppm which was less than the recommended limits of WHO. The cadmium concentration in soil samples was 0.2 ± 0.05 to 1.90 ± 1.40 ppm, higher than WHO's safe limits^7^. One study was conducted to assess the contamination of heavy metals in Iran and five east, west, south, north, and center stations were selected to sample drinking water. Samples were analyzed using (ICP-MS). The west and north sites were high in arsenic, nickel, mercury, and lead concentration. The east side was high in chromium concentration^8^. In 2019, a study was conducted on heavy metal assessment in China. Shanghai showed a lower concentration of cadmium in different mediums except for water. The highest mercury and lead concentrations were observed in Wuhan and Taiyuan, respectively^9^. A higher chromium concentration was observed in outdoor air, indoor air, traffic air and water in Taiyuan, Chengdu, Shanghai and Wuhan, respectively^10^.

The heavy metals can be removed from the contaminated sites using chemical, physical, and biological methods^11,12^. The chemical methods involved chemical precipitation, floatation, coagulation, ion exchange, and flocculation^13^. The physical methods involved granular activated carbon, membrane filtration, photocatalysis, soil washing, electrokinetic, and adsorption^14^. The biological methods used to remove or convert heavy metals into less toxic forms involved biotransformation, biosorption, and bioleaching^15^. Bioremediation uses microbes or microbial enzymes to remove metals from the environment. Bioremediation is a cost-effective and eco-friendly method that does not produce secondary pollutants^16^.

This study assessed heavy metals' concentration in industrial wastewater and was analyzed using atomic absorption spectroscopy. The main objective is to identify the cadmium resistance bacteria through 16S rRNA characterization, present in collected industrial samples. The cadmium-resistant *cadA* gene along with the disease-causing genes i.e., *tcpA* and *UreC* were identified through PCR amplification. In-silico studies were performed to edit or truncate identified virulent genes through a computational CRISPR cas 9 system. The gRNAs were selected based on their G.C. content and stability of the targeted disease-causing genes using CRISPOR and ENDMEMO, respectively. The expression analysis of the edited genes was determined via SnapGene. Subsequently, the identified genetically engineered bacteria can be used as the potential source for the bioaccumulation of cadmium from industrial wastewater.

## Materials and methods

**Figure 1:**
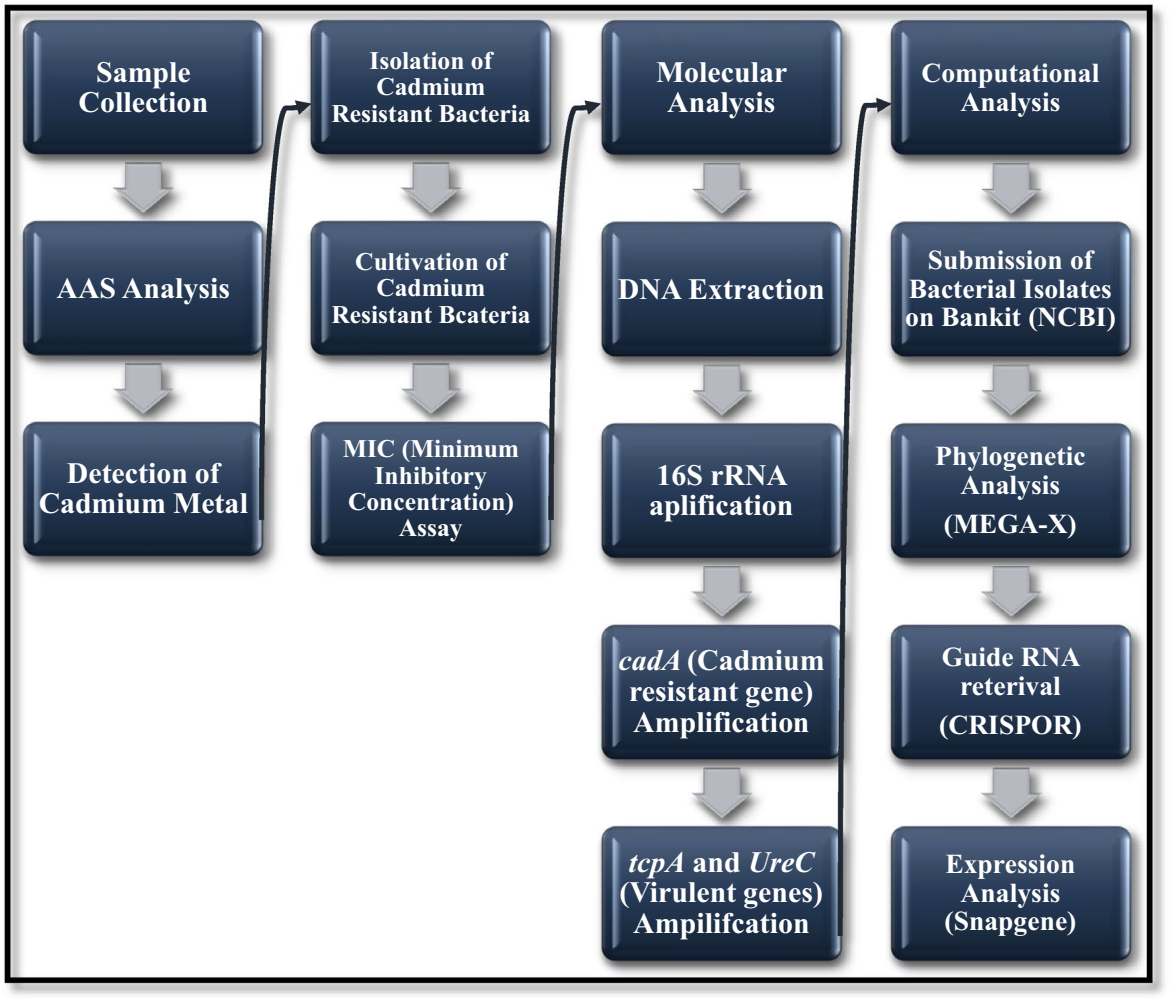
Flow chart of methodology.

This study was conducted from June 2021 to December 2021 in the Biotechnology lab, Microbiology lab and Molecular Biotechnology and bioinformatics lab of the University of Central Punjab (UCP). The complete flow chart of the methodology is given in Fig. 1.

### Sample collection

The wastewater samples were collected from the tanneries of Kasur, known as Pakistan's most significant industrial area. The collected samples belong to different treatment phases, including chrome-treated industrial wastewater, NaCl-treated industrial wastewater, and mixed industrial wastewater samples (IS1, IS2 and IS3).

### Atomic absorption spectroscopy of water samples

Atomic absorption spectroscopy (AAS) is an analytical technique used to determine the concentration of metal ions or atoms in a sample. The heavy metals concentration of industrial wastewater was detected by AAS. These heavy metals contaminate water, soil, and other resources, so atomic absorption spectroscopy can easily measure their presence.

### Isolation and cultivation of bacteria

To cultivate cadmium-resistant bacterial strains, the salt of cadmium chloride is used in Lysogeny broth (L.B.) and Muller Hinton media. The bacteria that resist cadmium can selectively grow on the medium. The growth medium was supplemented with the 50 ppm stress of cadmium salt.

### Suspension growth of resistant bacterial strains

The lysogeny broth (L.B.) was used, for suspension growth. L.B. was prepared by adding 2.5 g of tryptone, 1.25 g of yeast extract and 2.5 g of NaCl in 250 ml distilled water. The media was autoclaved at 121 °C temperature and 15 psi pressure for 45 min. The media was supplemented with 1.25 ml of cadmium chloride salt to give 50 ppm stress for the selective growth of cadmium resistant strains. The media supplemented with CdCl_2_ was poured into 50 ml falcon tubes and labelled the tubes as IS1, IS2 and IS3. With the help of a sterilized loop, the water samples were inoculated into each falcon tube. Afterward, all the tubes were kept in an incubator shaker at 37 °C at the speed of 180 rpm for overnight suspension growth. The suspension growth was seen in each of the tubes, after 24 h.

### Inoculation of microbes

The overnight grown suspension of bacteria was further inoculated on Petri plates using (M.H.) agar. The media was prepared by adding 250 ml distilled water and 9.5 g of M.H. agar into a 250 ml conical flask. The media was autoclaved at 121 °C and 15 Psi for 45 min. Before pouring, 1.25 ml of the Muller Hinton (M.H) media was taken out, and the same amount of CdCl_2_ stock solution was added to give the 50 ppm stress to get pure isolates of cadmium resistant strains. After adding cadmium salt, the media was poured into autoclaved Petri plates in a laminar flow hood. The plates were labelled as IS1, IS2 and IS3. After the media solidified, the inoculum loop was suspended in previously enriched L.B. containing cadmium resistant bacteria. The loop was streaked into solidified M.H. plates and held at 37 °C in an incubator overnight. After 24 h, fine growth or isolated colonies were seen. For molecular analysis and DNA extraction, the single colony was inoculated overnight into L.B. broth from each plate to get the suspension of cadmium resistant strains.

### MIC assay to evaluate cadmium tolerance of IS1, IS2 and IS3

The stock solution of 100 g/L was prepared using 1.63 g of cadmium chloride and autoclaved distilled water. The lysogeny broth (L.B.) broth media was prepared using 2.5 g tryptone, 1.25 g yeast extract and 2.5 g NaCl for 250 ml. After autoclaving, the media was supplemented with cadmium. The tolerance level is calculated; all the bacterial isolates were grown on 200 ppm, 400 ppm, 600 ppm, 800 ppm and 1000 ppm cadmium stress in Lysogeny broth. The L.B. media was inoculated with overnight grown bacterial suspension and kept on an incubator shaker for 5–6 days at 180 rpm and 37 °C. After 6 days, the optical density of each inoculated broth media was measured at 600 nm on a spectrophotometer.

### Molecular analysis for the identification of bacterial isolates

The genomic DNA from each sample strain was extracted using the CTAB method followed by 16S rRNA amplification. The main three were followed for the DNA extraction including lysis, precipitation, washing and purification. In the bacterial DNA extraction using the CTAB method, bacterial cells undergo lysis with a CTAB buffer comprising 2–5% cetyltrimethylammonium bromide, Tris–HCl, and EDTA, with the addition of proteinase K for protein digestion. Subsequent DNA extraction involves the use of Phenol–Chloroform-Isoamyl Alcohol (PCI), DNA precipitation with isopropanol, and purification through ethanol washing. The resulting DNA pellet is then resuspended in an appropriate buffer. For gel electrophoresis, an agarose gel (1%) in TAE buffer is prepared, and DNA samples, mixed with loading dye, are loaded alongside a DNA ladder for size estimation. Gel electrophoresis is conducted at 90 V until sufficient separation is achieved. The gel casing, including the agarose concentration, is determined based on the desired size range of DNA fragments. It separates the DNA on the base of molecular weight. Staining with ethidium bromide enables visualization, and gel images are captured using a gel documentation system equipped with a UV transilluminator and a camera.

### Polymerase Chain reaction for 16S rRNA amplification

PCR is a thermocycler process that works on the principle of temperature gradient under optimized reaction conditions. The 16S rRNA amplification carries out the identification of bacterial strains. 9F and 1500R primers were used to amplify and identify unknown bacterial strains given in Table 1. The PCR reaction is prepared in autoclaved PCR tubes by adding 12.5 µl master mix, 1 µ forward primer, 1 µl reverse primer, 2 µl template DNA and 12.5 µl double distilled water. The temperature profile to amplify 16S rRNA was followed by initial denaturation of 95 °C for 5 min, second denaturation of 94 °C for 1 min, annealing at 55 °C for 1 min, the extension of 72 °C for 1 min, the final extension of 72 °C for 7 min and storage for 12 min on 4 °C. PCR runs for 35 cycles for the amplification of 16S rRNA. After PCR amplification, amplicons were resolved on gel electrophoresis using the ladder of 1 Kb. Samples were sent for sequencing to Xbase sequencing company, Malaysia, through ABI (Applied Bioscience International). Sequencing confirms which bacterial strains can tolerate the stress of cadmium.

**Table 1:** Primer sequences of genes.

Gene	Primers	Sequence	Amplicon size (bp)	Primer Tm (°C)
16S rRNA	Forward	5′–GAGTTTGATCCTGGCTCAG–3′	1490	55
	Reverse	3′–GGCTACCTTGTTACGA–5′		
*cadA*	Forward	5′–ATCACCATACCGGAAGCTGC–3′	623	57
	Reverse	3′–TAGTGACCAGCAGCAGGCAG–5′		
*tcpA*	Forward	5′–GAAGAAGTTTGTAAAAGAAGAACAC–3′	472	55.5
	Reverse	3′–GAAAGGACCTTCTTTCACGTTG–5′^17,18^		
*UreC*	Forward	5′–GTTATTCGTGATGGTATGGG–3′	317	57
	Reverse	3′–GTAAAGGTGGTTACGCCAGA–5′^19^		

### PCR Amplification of *cadA, tcpA *and *UreC* genes

All the bacterial species, including IS 1, IS 2 and IS 3, were amplified for the presence of cadmium resistant gene *cadA*. The *tcpA* and *UreC* gene is responsible for causing cholera and urinary tract infections in humans. Cadmium resistance and disease causing *tcpA* and *UreC* genes of bacteria show an inverse relationship between them. The primers of all the three genes were designed manually by the bioinformatics tool named MSA (multiple sequence alignment)/ clustal W provided in Table 1. The sequence of all the primers was sent to MACROGEN KOREA for primer designing. The stock solution of primers was prepared by adding nuclease-free water. The working solution was prepared by adding 90ul double distilled water and 10ul stock of the primer. The working solution of the primers was used for the amplification of genes.

### Sequence submission on Bankit (NCBI)

The 16S rRNA were submitted on Bankit (NCBI) (https://www.ncbi.nlm.nih.gov/WebSub/) to get the specific accession number for the bacterial isolates.

### Phylogenetic analysis

MEGA-X is software used to understand the evolutionary relationship between different bacterial strains. The evolutionary analysis was conducted using the neighbor-joining method at 1000 bootstrap value for each analysis.

### Designing gRNA and evaluation of G.C. content

The single-stranded guided RNA was designed using the tool CRISPOR (http://crispor.tefor.net/). The G.C. contents of the gRNA of the *UreC* and *tcpA* gene were analyzed using ENDMEMO (http://endmemo.com/). This tool predicted the G.C. content based on an online G.C. content plot. Guanine and cytosine show a strong bonding based on a triple hydrogen bond. The G.C. content of more than 50% makes the stable RNA–DNA duplex when ligating into the cloning vector. The gRNA of the *UreC* gene and *tcpA* gene was selected based on the off-targets and G.C. content.

### Assembling of gRNA in expression vector

After selecting desired RNA guides for *UreC* and *tcpA*, they were assembled into an expression vector. The ligation of guiding RNA into an expression vector makes it genetically modified. For genetic modification, the *UreC* gRNA and *tcpA* gRNA were assembled into lenti CRISPR v2 individually. The lenti CRISPR v2 was selected because it has filler regions, BsmBI restriction site, promoter regions and cas9.

## Results

### Heavy metals concentration in water samples

Atomic absorption spectrometry (AAS) is a reliable process of detecting heavy metals and metalloids in different environmental mediums, mainly water^20^. The concentration of heavy metals in IS1, IS2 and IS3 was calculated using atomic absorption spectroscopy (AAS) given in Table 2.

**Table 2:** Cadmium concentration evaluated in industrial wastewater using atomic absorption spectroscopy.

WHO Recommended cadmium concentration	0.003 ppm		
Samples	IS1	IS2	IS3
Cadmium concentration (ppm)	29.6	52	76.4

### Inoculation of bacterial isolates

**Figure 2:**
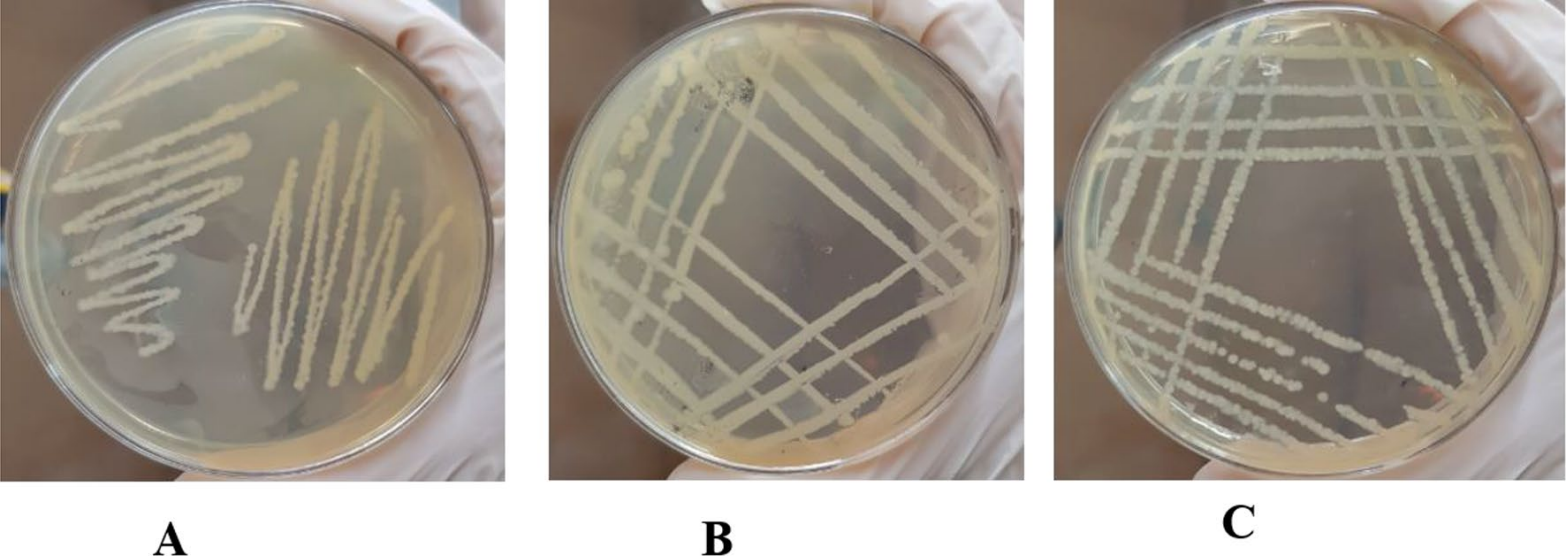
Growth of Pure Bacterial Isolates in 50 ppm Cadmium salt stress (**A**) Vibrio cholerae, (**B**) Proteus mirabilis and (**C**) Vibrio metoecus.

The suspension of bacterial growth was further streaked on M.H. agar plates containing 1.63 g/L cadmium chloride salt. The agar plates were kept at 37 °C overnight for the growth of pure isolates shown in Fig. 2. Plate A represents the pure isolates of the bacteria from IS1 (*Vibrio cholerae*), Plate B represents the pure isolates of the bacteria from IS2 (*Proteus mirabilis*), and Plate C represents the pure isolates from sample IS3 (*Vibrio metoecus*).

### MIC for the evaluation of resistance against cadmium

**Figure 3:**
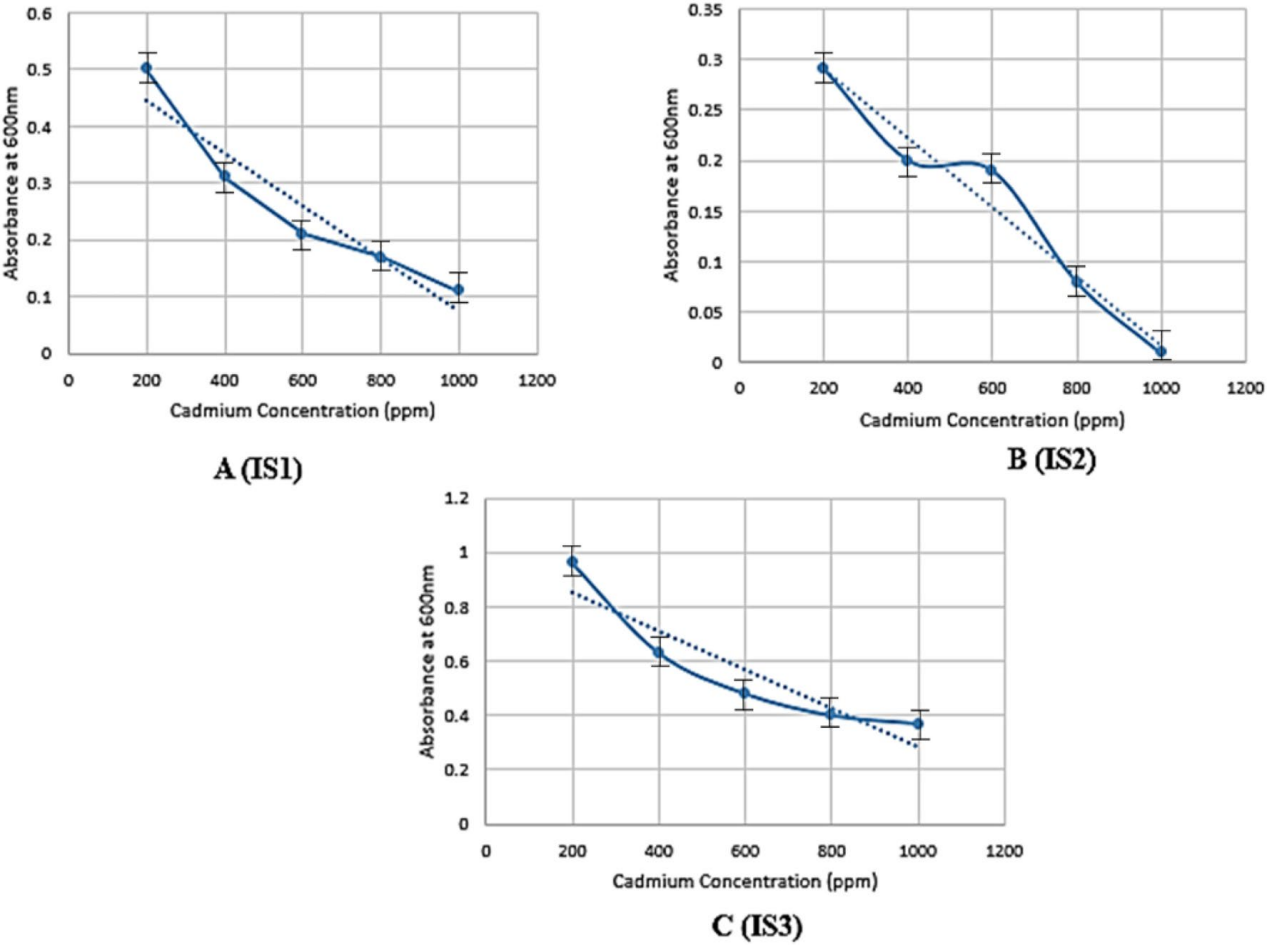
Determination of Minimum Inhibitory Concentration of Cadmium by (**A**) IS1 (Vibrio cholerae), (**B**) IS2 (Proteus mirabilis) and (**C**) IS3 (Vibrio metoecus) measured 600 nm.

All bacterial isolates' minimum inhibitory concentration was measured to determine cadmium degradation. The test indicated that the maximum tolerance of cadmium was up to 1000 ppm for the concentration of 1.63 g/L. Bacteria that have resistance capacity will use cadmium and other nutrients to grow. The optical density was decreased with the increased concentration of cadmium. The presence of cadmium in the cell indicates its toxic effect on the bacterial isolates. The bacterial isolates adapted to a polluted cadmium environment might change their morphology, function, and activation of resistant factors or genes. In the case of MIC, the bacteria which can resist the elevated level of cadmium may be helpful for the biosorption (bioaccumulation) of cadmium from industrial wastewater shown in Fig. 3A–C.

### Molecular characterization

**Figure 4:**
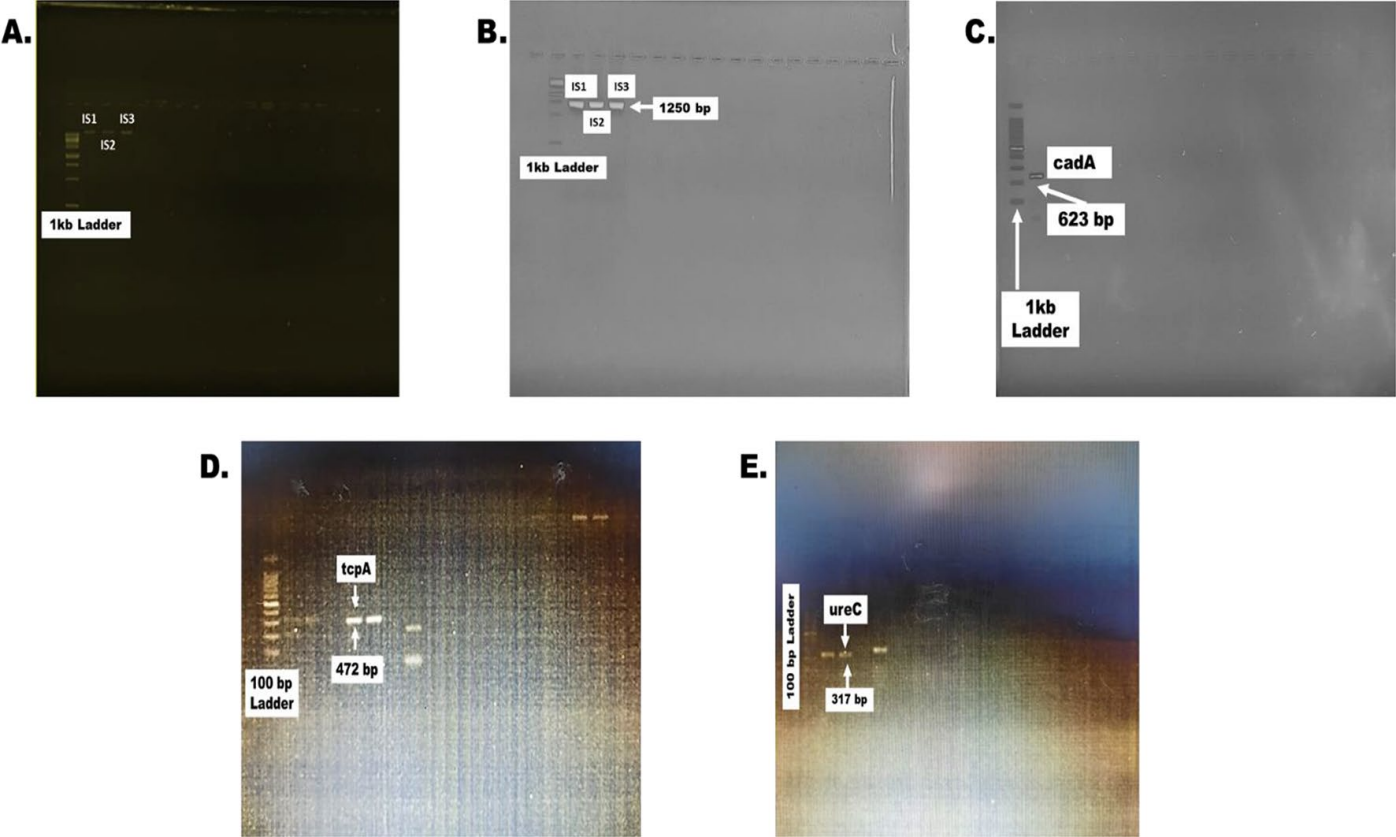
Visualization of amplicons using GEL DOCUMENTATION SYSTEM (**A**) Bacterial Genomic DNA of Cadmium Resistant Bacteria, (**B**) 16S rRNA amplification of extracted DNA (1250 bp), (**C**) Cadmium Resistant CadA gene visualization of amplicons (623 bp) (**D**) Cholera causing tcpA gene visualization of amplicons (472 bp), (**E**) Urinary Tract Infection (UTI) causing UreC gene visualization of amplicons (317 bp).

The extracted DNA was visualized under the UV-documentation system after gel electrophoresis. 1% agarose gel was used at 90 V for 45 min to visualize DNA bands. After 45 min, the DNA bands were seen under UVB on the GEL DOCUMENTATION system, as seen in Fig. 4A.

### 16S rRNA amplification of cadmium resistant bacterial DNA

All the three samples were amplified for the 16S rRNA, and the bands were seen using 2% gel on the GEL DOCUMENTATION system. For the confirmation of size, a 1 Kb ladder was used. The size of the amplicons was 1450 bp, as given in Fig. 4B. After amplifying the 16S ribosomal RNA gene, all the samples were sent for sequencing from ABI.

### Amplification of cadmium resistant and virulence genes

Cadmium resistant *cadA* gene was amplified in *Vibrio spp* and *Proteus spp* with the product size of 623 bp given in Fig. 4C. The Cholera causing *tcpA* gene was amplified in *Vibrio cholerae* with the product size of 472 bp shown in Fig. 4D. The 100 bp ladder was used, and bands were visualized under UV GEL. The urinary tract infection (UTI) infection-causing gene was amplified in *Proteus mirabilis* with the product size of 317 bp given in Fig. 4E. The 100 bp ladder was used, and bands were visualized under the UV GEL DOCUMENTATION system.

### Accession ID’s of bacterial isolates

After the sequencing of the 16S rRNA gene, nucleotide sequences were obtained for each of the samples. The identified bacterial isolates were submitted on Bankit (NCBI) and were assigned a specific accession number as all the sequences were unique in nature with a query coverage of 99% (Table 3).

**Table 3:** Sequence submission of isolated bacterial identified through 16S rRNA amplification.

Bacterial isolates	Strain ID on Bankit	Assigned accession number	Bacterial name	Query coverage (%)	Percentage identity (%)	E-value
IS1	MBBL16	OL989225.1	*Vibrio cholerae*	99	95.13	0.0
IS2	MBBL17	OL989227.1	*Proteus mirabilis*	99	96.42	0.0
IS3	MBBL18	OL989229.1	*Vibrio metoecus*	99	95.45	0.0

### Phylogenetic analysis

**Figure 5:**
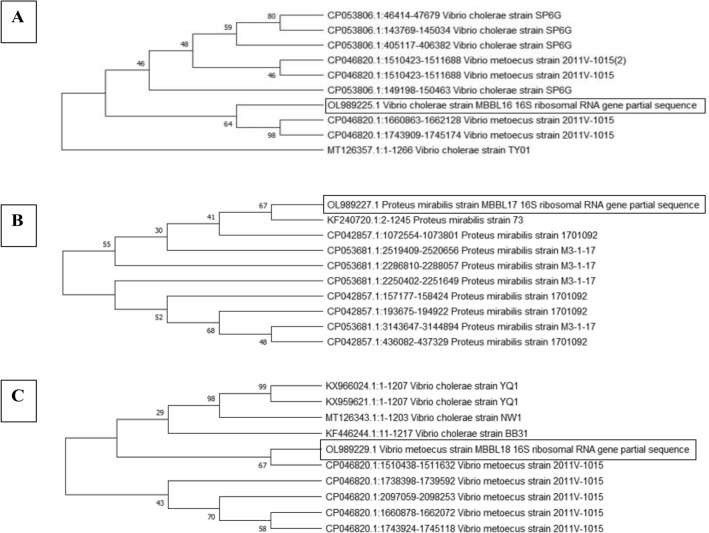
Phylogenetic tree illustration of (**A**) *Vibrio Cholerae*, (**B**) *Proteus mirabilis*, (**C**) *Proteus mirabilis* by Neighbor-Joining Method at 1000 bootstrap value.

The MBBL16 *Vibrio cholerae* showed homology with *Vibrio metoecus* 2011V-1015 with the bootstrap value of 64, and the out group is MT126357.1:1-1266 *Vibrio Cholera* strain TY01 shown in Fig. 5A. The MBBL17 *Proteus mirabilis* showed the sequence homology with *Proteus mirabilis* strain 73 with the bootstrap value of 67 and outgroup is CP053681.1:2250402–2251649 *Proteus mirabilis* M3-1-17 shown in Fig. 5B. The MBBL18 *Vibrio metoecus* showed the sequence homology with *Vibrio metoecus* strain 2011V-1015 with a bootstrap value of 67, and the out group is CP046820.1:1738398–1739592 *Proteus mirabilis* Strain 2011V-1015 shown in Fig. 5C.

### Guide RNA retrieval for *UreC* and *tcpA*

**Figure 6:**
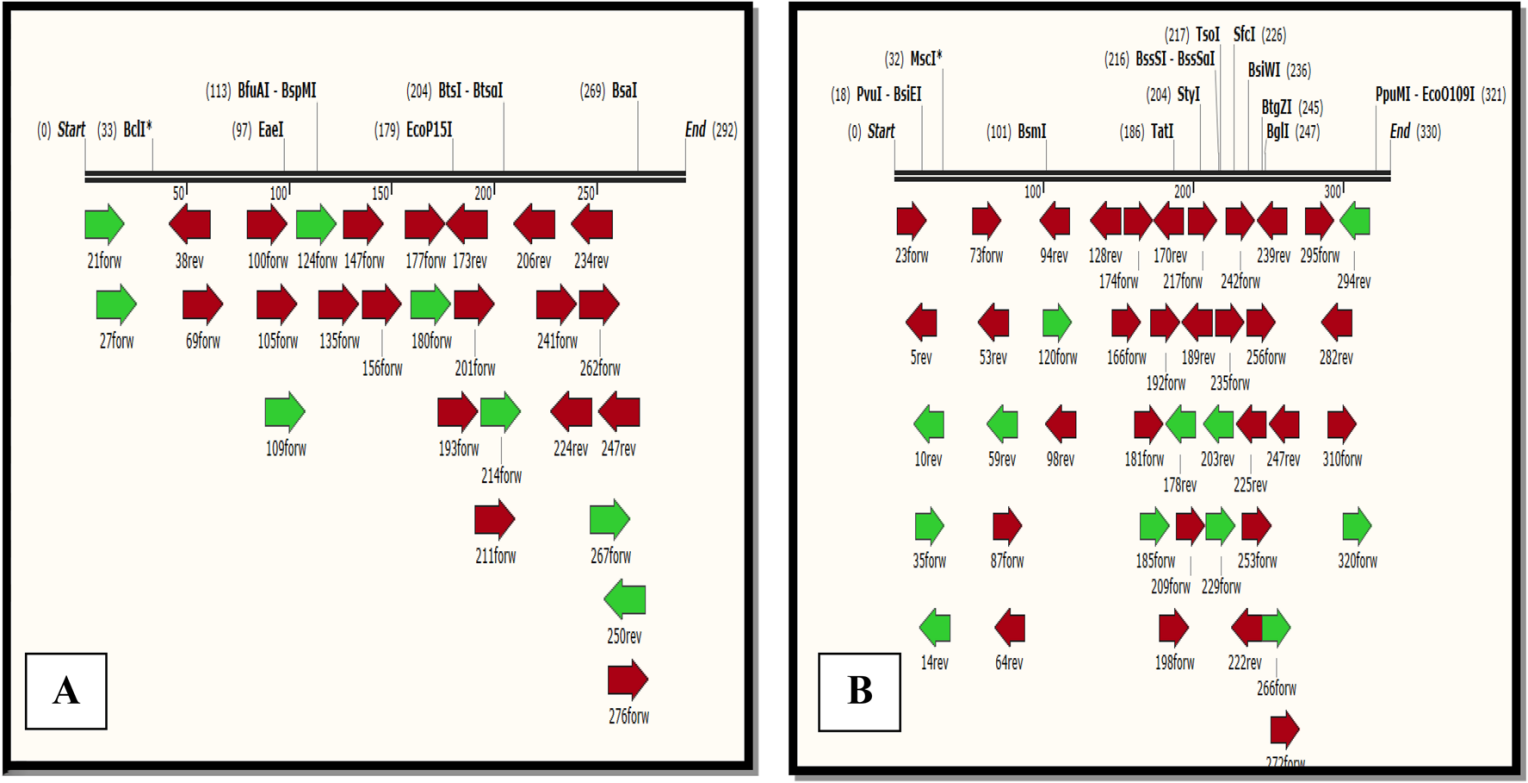
Possible gRNA sequences generated by CRISPOR (**A**) UreC (Urease subunit alpha) gene and (**B**) tcpA (Toxin coregulated pilin precursor) gene.

**Figure 7:**
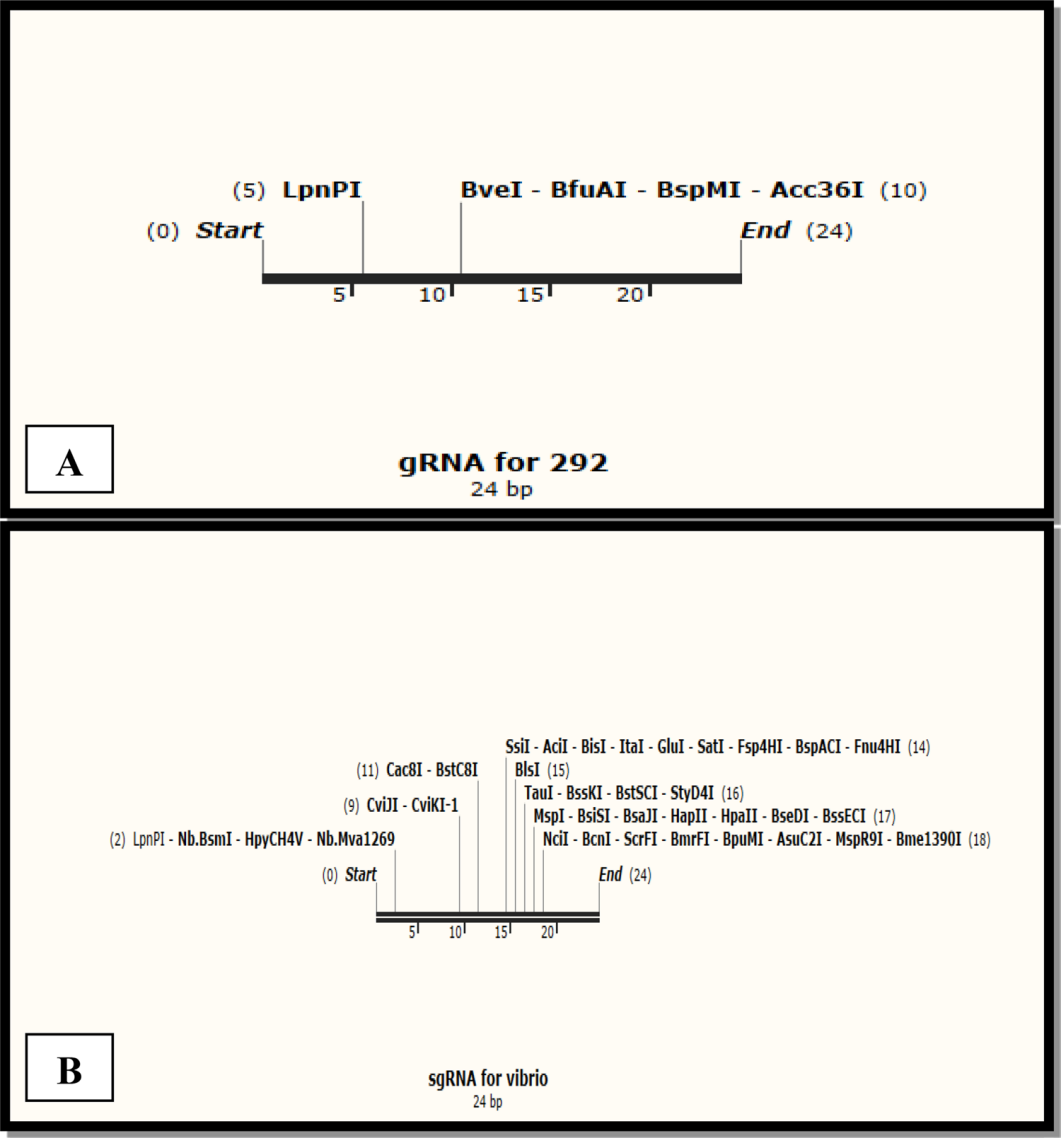
Selected gRNA of (**A**) *UreC* gene and (**B**) *tcpA* gene for analysis.

The UreC gene generated 27 gRNA sequences, from which 5 were selected to incorporate into the expression vector. The possible generated gRNAs for UreC are given in Fig. 6A. The green color indicates the high specificity in the genome, while red indicates PAM's guide sequence's low specificity. From five selected gRNAs, 124 forward was the best way to ligate into the filler region given in Table 4. Reverse gRNA was neglected because it did not fit into filler regions. The gRNA sequence of 124 forward along with restriction sites is provided in Fig. 7A. The tcpA gene generated a total of 42 gRNA sequences, from which 8 were selected for incorporation into the expression vector. The possible generated gRNAs for tcpA are given in Fig. 6B. From eight selected gRNAs, 120 forward was the best way to ligate into the filler region given in Table 4. Other forward and reverse gRNAs are neglected because of their inability to fit into the filler region. The gRNA sequence of 120 forward restriction sites is given in Fig. 7B.

**Table 4:** Selected gRNA of *UreC* and *tcpA* genes for CRISPR based editing.

gRNA	Target sequences	MIT Score	Off-target counts
UreC			
27forward	GTTTTGTGCTGAGTGTGTCGATGT	100	1
109forward	ATTAAAGATGGCCGTATTGTCGGT	100	1
124forward	ATTGTCGGTATTGGCAAGGCAGGT	100	1
180forward	CATTGGCCCCGGAACAGAAGTTGT	100	1
214forward	CATTGGCCCCGGAACAGAAGTTGT	100	1
tcpA			
35forward	CGATCGTGTGGTCATTGGCCAGGT	100	1
120forward	TGCATTCGGCTCGCCGCCGGGGGT	100	1
185forward	CCCGTACTTTTGTACGGGGTTTGT	100	1
229forward	GGGTCAGCTCGTGACAACTATAGT	100	1

### Calculated G.C. content

The calculated G.C. content of gRNAs of *UreC* 124 forward is 50% with 24 bp length, and given the G.C. content for gRNA of *tcpA* 120 forward is 75% with 24 bp length.

### Cloning of gRNA into expression vector

**Figure 8:**
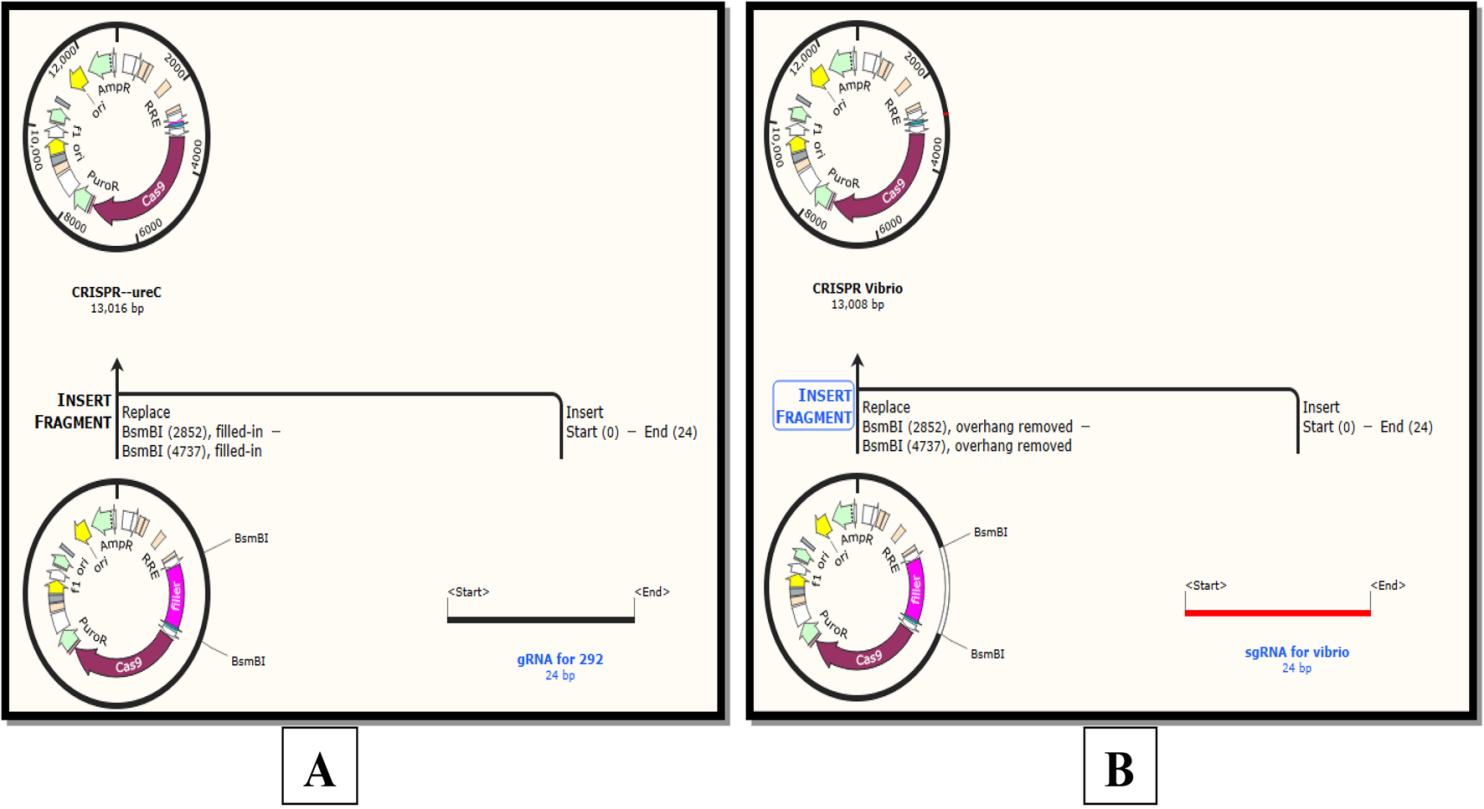
(**A**) Expression of 124forw gRNA of UreC into Expression vector lentiCRISPR v2 and (**B**) Expression of 120forw gRNA of tcpA into Expression vector lentiCRISPR v2.

The expression vector was retrieved from an offline tool SnapGene. The vector originates from replication, CMV region, cas9 that generates RNA-guided double-stranded breaks in DNA, EM7 promoter (synthetic bacterial promoter), gRNA scaffold, filler region and several antibiotic-resistant promoters. The filler region allows the gRNA to incorporate into an expression vector. After digestion with the restriction enzyme BsmBI, the gRNA of UreC and tcpA can be cloned into the expression vector. The designed gRNA of 24 bp for UreC was cloned into lentiCRISPR v2 (14,873 bp), shown in Fig. 8A. The desired gRNA of tcpA was cloned into the expression lentiCRISPR v2 (14,873) shown in Fig. 8B. After transforming expression vectors containing guide RNAs for the UreC gene and tcpA gene, these specific virulent bacterial genes can be removed from *Vibrio cholera* and *Proteus mirabilis* by the activity of cas9. The bacteria are said to be genetically modified cadmium resistant.

## Discussion

In the last century, urbanization has increased, which is directly involved in the growth of industrialization^21^. Industries' waste products can produce many pollutants and chemicals, including heavy metals, organic pollutants, inorganic ions, organometallic compounds, radioactive isotopes, and gaseous pollutants that highly contaminate the atmosphere, water, and land^22^. The increase in industrial pollutants can cause serious health effects on humans, plants, and marine animals. Heavy metals that cause serious health effects involve cadmium, chromium, arsenic, cadmium, lead, mercury, zinc etc.^23^. The safe limits of cadmium in wastewater, drinking water, plants and soil are 0.003 ppm, 0.005 ppm, 0.02 ppm, and 0.003 ppm, respectively^7^. In this study, the cadmium concentration in industrial wastewater has a range of 29.6–76.4 ppm, which is immensely high compared to safe limits recommended by WHO. The safe limits of chromium in wastewater, drinking water, plants and soil is 0.05 ppm, 0.1 ppm, 1.3 ppm, and 0.1 ppm, respectively^7^. In this study, the concentration of chromium in industrial wastewater is up to 1316.5 ppm, which is much higher than the safe limits recommended by WHO. The safe limits of mercury in wastewater, drinking water, plants and soil are 0.001 ppm, 0.006 ppm, 0.1 ppm, and − 0.08 ppm, respectively. In this study, mercury concentration in industrial wastewater was not detected.

The safe limits of lead in wastewater, drinking water, plants and soil is 0.01 ppm, 0.01 ppm, 0.1–0.3 ppm, and 0.1 ppm, respectively^7^. In this study, the lead concentration in industrial wastewater ranges from 5 to 7.4 ppm, which is high compared to the safe limits recommended by WHO. The safe limit of arsenic in water is 0.01 ppm recommended by WHO^24^. In this study, the concentration of arsenic in industrial wastewater ranges from 2 to 16 ppm, which is highly toxic compared to the safe limits recommended by WHO. Cadmium metals in industrial wastewater were detected by atomic absorption spectroscopy. Heavy metals can be removed from the environment through chemical, physical, and biological methods^25,26^. All other methods except biological methods produce secondary pollutants and are expensive. Biological methods do not produce any secondary pollutants in the environment^27^. Removing heavy metals through bacteria and their metabolites is an eco-friendly and cost-effective way^28,29^. Previously, *Alcaligenes faecalis, Pseudomonas aeruginosa, Bacillus pumilus* and *Brevibacterium iodinium* have been used to remove mercury^30^. *Brevibacterium spp* and *Bacillus pumilus* showed the potential to remove a high amount of lead from the environment^31^. *Bacillus subtilis, Bacillus megaterium, Aspergillus niger, and Penicillium* sp showed the bio-sorption capacity against cadmium, lead and chromium^32^.

The 16S rRNA cadmium resistant strains involved *Vibrio choloerae* (MBBL16), which was isolated from IS1*, Proteus mirabilis* (MBBL17), which was isolated from IS2 and *Vibrio metoecus* (MBBL18) which was isolated from IS3. All the cadmium degrading strains have some virulent effect. The *Vibrio cholerae* and *Vibrio metoecus* are virulent strains that can cause severe cholera. The *Proteus mirabilis* is also a bacterial strain that can cause severe urinary tract infection (UTI), especially in women. *Vibrio cholerae and Proteus mirabilis* showed sensitivity in response to several antibiotics by forming clear zone involving meropenem, norfloxacin, and amikacin. *P.mirabillis* and *V.cholerae* showed resistance against Colistin. *V.metoecus* showed antibiotic sensitivity in response to all five antibiotics by forming clear zones. All virulent bacterial strains were sensitive and resistant to antibiotics and conferring bio-sorption of cadmium. The cadmium resistant *cadA* gene was amplified in *Vibrio spp* and *Proteus spp,* which confirmed their ability of cadmium removal on a genetic level. The *tcpA* (toxin coregulated pilin precursor) was identified as a virulent gene of *Vibrio cholerae* with the size of 472 bp. In contrast, *UreC* (urease subunit alpha) was identified as the virulent gene of *Proteus mirabilis* with 317 bp. To remove cadmium from industrial wastewater, it is necessary to remove the virulence mechanism of *Vibrio cholerae, and Vibrio metoecus.*

*In-silico* analyses were performed to remove the virulent genes of urinary tract infection and cholera. The gRNAs of both genes were retrieved from CRISPOR. Guiding RNA was selected based on more than 50% of the G.C. content. G.C. content was calculated from ENDMEMO. The specificity of gRNA. The gRNA of 124 forw was selected from the UreC suitable for the filler region, while the gRNA of 120forw was selected from tcpA, which was most suitable for the filler region. The expression vector named lentiCRISPR v2 was retrieved from SnapGene. LentiCRISPR v2 was selected due to its various restriction sites and the presence of cas9. The expression vector was first cut by restriction enzyme BsmBI and ligated with the gRNA of UreC and tcpA. The cas9 in lentiCRISPR v2 allowed cutting the gRNA sequence from the bacterial genome. After the sequence was truncated, the function of the virulent gene of UreC and tcpA was stopped. The current study is lacking the biological mechanisms employed by cadmium-resistant strains and the potential environmental impact of introducing modified bacteria are not deeply explored. Furthermore, the study lacks detailed data presentation, such as the total volume or flow rate of wastewater, essential for understanding contaminant loads. The regulatory and policy implications of heavy metal concentrations exceeding recommended limits are also not addressed. Addressing these limitations through more comprehensive experimental validations.

## Conclusion

Anthropogenic activities and industrialization increase environmental contamination. Heavy metals affect the deposition in water, soil, land, marine animals, humans, and plants. The present study will help us to fight against the heavy metals present in the contaminated water. Moreover, amplification of the cadA gene proved that *Vibrio* and *Proteus Proteus mirabilis* are cadmium resistant. Amplification of tcpA and UreC showed virulence and resistant genes in cadmium-resistant bacteria. CRISPOR predicted virulent strains' gRNAs and BsmBI-restricted lentiCRISPR v2 through SnapGene. SnapGene was used to express lentiCRISPR v2 gRNAs for UreC and tcpA. The production of gRNA in the vector allowed us to extract the cholera-causing tcpA gene and UTI-causing UreC gene from virulent bacterial strains and use them for cadmium bioaccumulation and removal. CRISPR-Cas9 will let us shorten disease-causing genes and alter pathogenic microorganisms for good. The gene-editing of the disease-causing bacteria will help in the future to use them to remove cadmium. CRISPR-cas9 gene editing mechanism allowed the use of even virulent bacterial strains to manage the toxic effects of heavy metals in the environment (Supplementary file).

### Supplementary Information

Supplementary Information.

## Data Availability

All the data has been included in the manuscript.
