# Supplementary material for: Molecular identification of Proteus mirabilis, Vibrio species leading to CRISPR-Cas9 modification of tcpA and UreC genes causing cholera and UTI
Source: Sci Rep. 2024 Apr 12;14:8563. doi: 10.1038/s41598-024-59340-9 (PMC11014924; doi:10.1038/s41598-024-59340-9)
Supplement: Supplementary file 1 — Supplementary Information. [file 41598_2024_59340_MOESM1_ESM.docx]

**SUPPLEMENTARY FIGURES OF GEL**

**
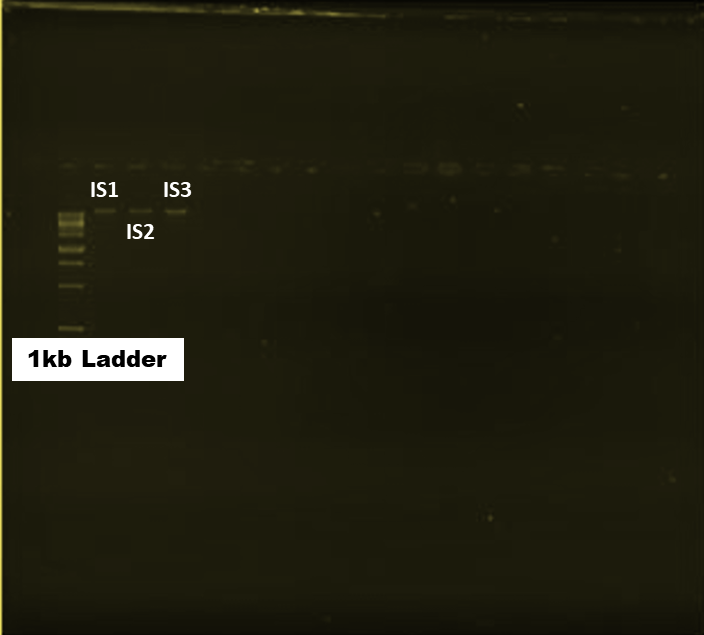
A.**

**
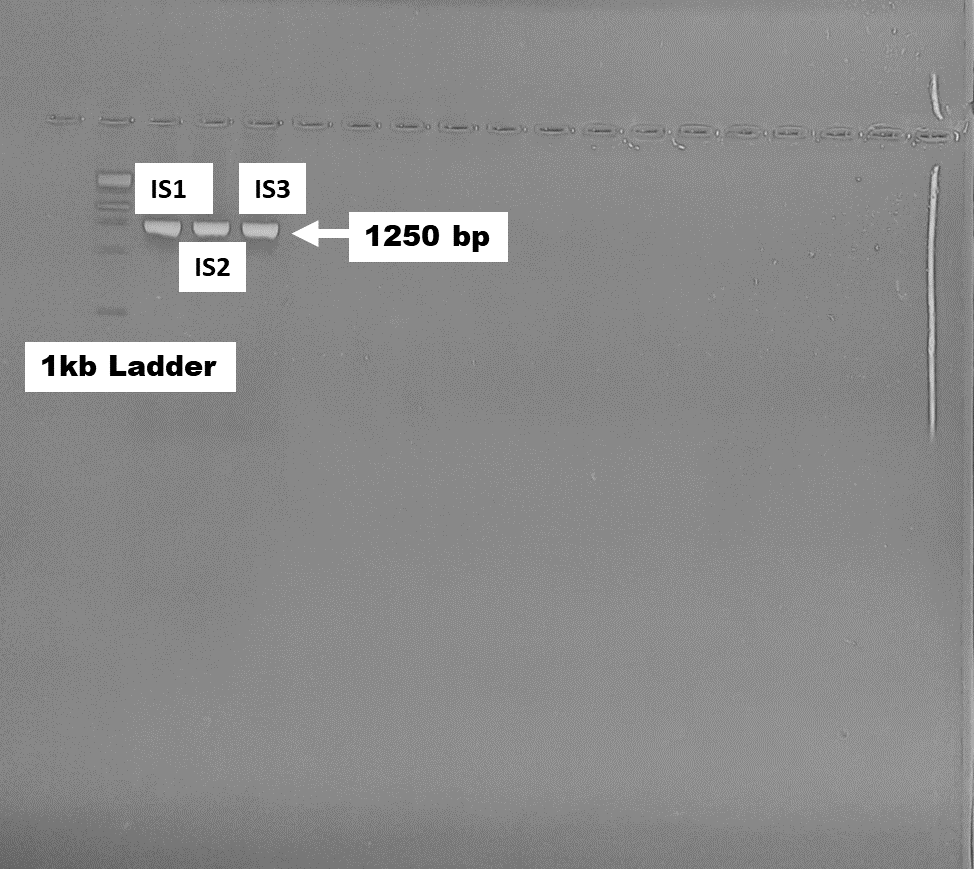
B.**

**
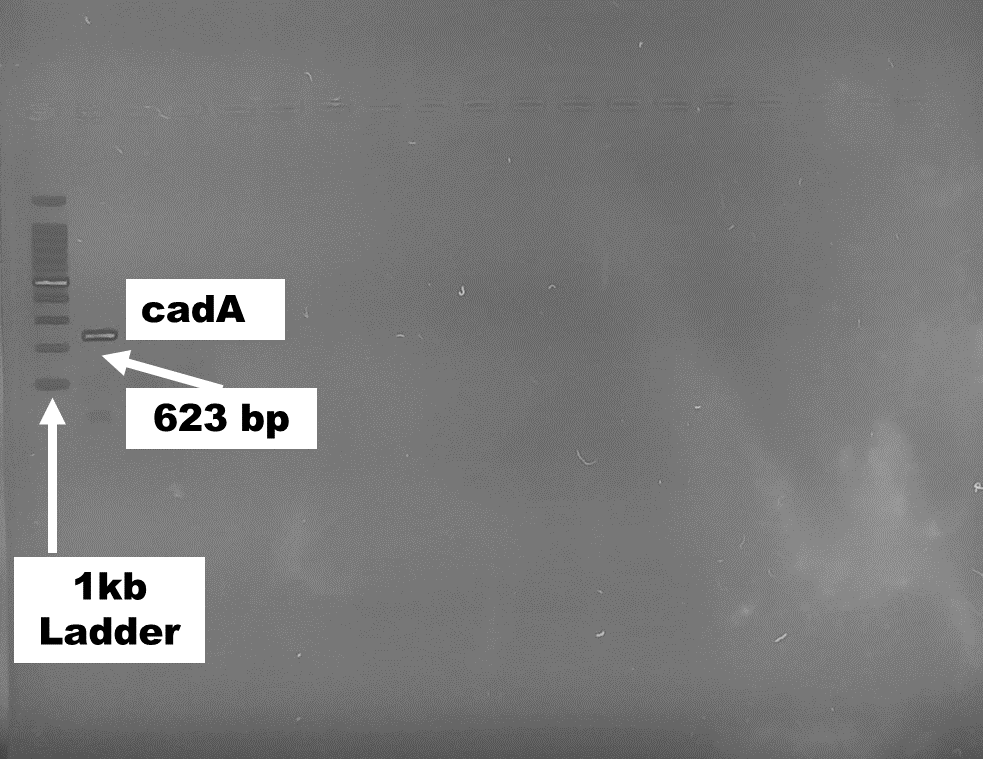
C.**

**
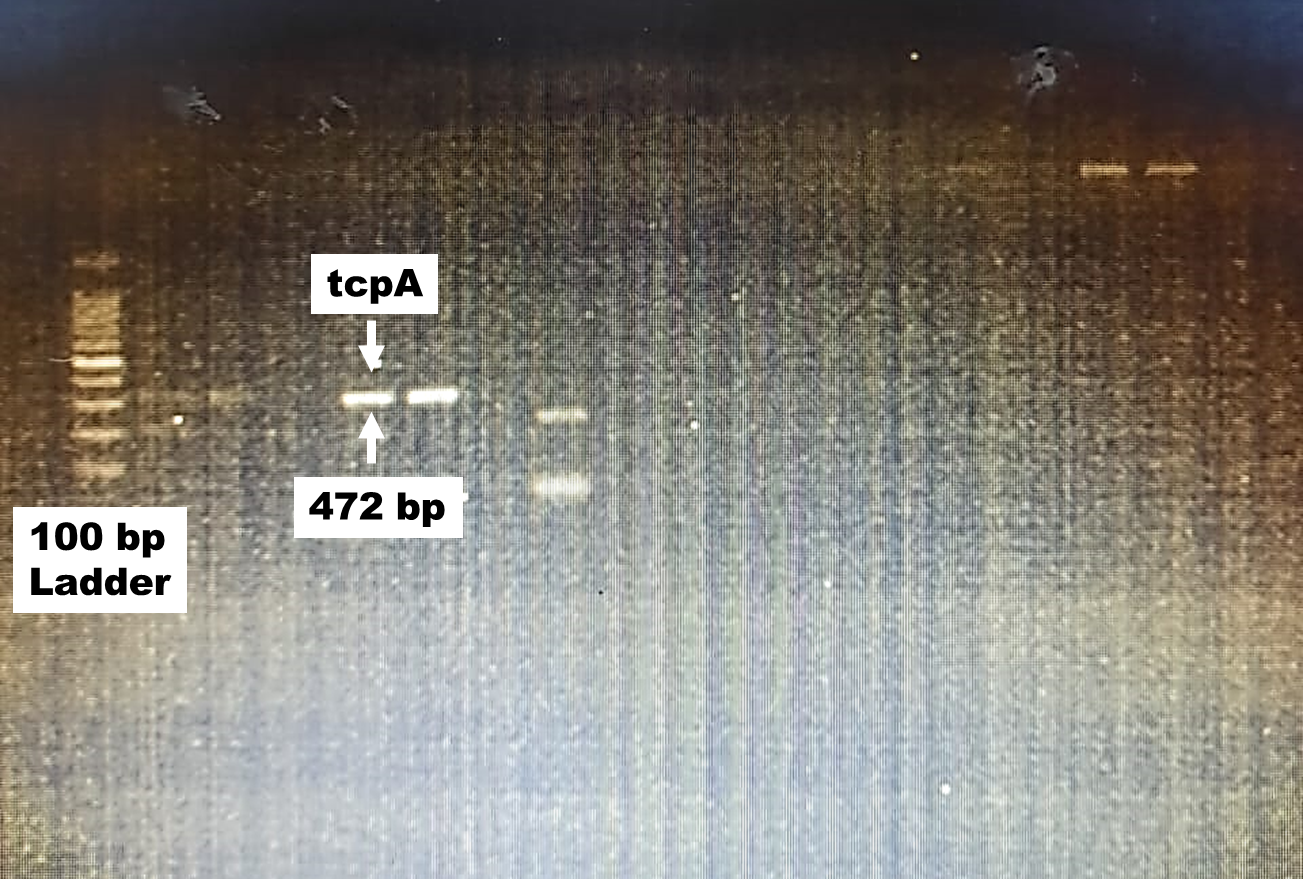
D.**

**
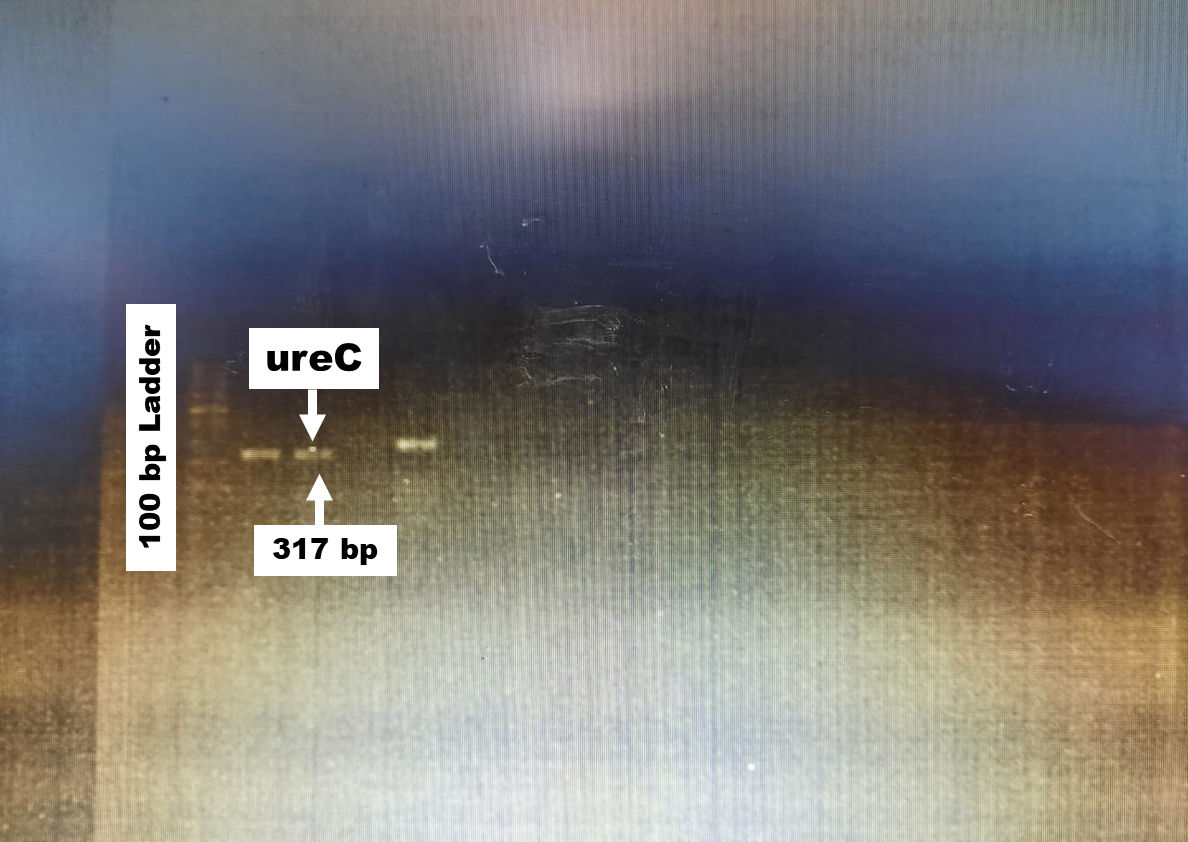
E.**

**Figure S1: Visualization of amplicons using GEL DOCUMENTATION SYSTEM A: Bacterial Genomic DNA of Cadmium Resistant Bacteria, B: 16S rRNA amplification of extracted DNA (1250bp), C: Cadmium Resistant CadA gene visualization of amplicons (623bp) D: Cholera causing tcpA gene visualization of amplicons (472bp), E: Urinary Tract Infection (UTI) causing UreC gene visualization of amplicons (317bp).**
